# The vegetable section of *Běnc $\overset{ˇ}{a}$ o Gāngmù*: classification architecture, taxonomic logic, and nomenclatural practice

**DOI:** 10.3389/fphar.2026.1946442

**Published:** 2026-09-21

**Authors:** Tung-Hu Tsai

**Affiliations:** 1 School of Chinese Medicine, College of Medicine, National Yang Ming Chiao Tung University, Taipei, Taiwan; 2 Institute of Traditional Medicine, College of Medicine, National Yang Ming Chiao Tung University, Taipei, Taiwan

**Keywords:** Běncǎo Gāngmù, botanical classification, nomenclature, taxonomic history, traditional Chinese medicine, vegetable section

## Abstract

The *Běnc $\overset{ˇ}{a}$ o Gāngmù* (Compendium of Materia Medica, 1596) by L $\overset{ˇ}{ı}$ Shízhēn (1518–1593) introduced a five-class botanical classification and nomenclature system for its vegetable section (Cài Bù, volumes 26–28), replacing the three-grade quality hierarchy that had organized all earlier Chinese pharmacopeias and marking a departure in the taxonomic history of Chinese natural history. The five classes—Hūnxīn Lèi (pungent aromatic, 32 taxa), Róuhuá Lèi (soft and smooth, 41 taxa), Lu $\overset{ˇ}{o}$ cài Lèi (fruit vegetables, 11 taxa), Shu $\overset{ˇ}{ı}$ cài Lèi (aquatic vegetables, 6 taxa), and Zhī'ěr Lèi (fungi and mushrooms, 15 taxa)—are distinguished by compound sensory, morphological, and ecological criteria and together comprise 105 edible taxa. This review, grounded in the ethnobotany of traditional Chinese medicine, examines the intellectual foundations of this classification system, its criteria and internal logic, its taxonomic apparatus (nomenclature conventions, etymology, synonymy, and inter-section transfers), and its relationship to earlier Chinese natural history traditions and the contemporaneous emergence of Linnaean systematic botany in Europe. Particular attention is given to the classification of fungi within the vegetable section, the documentation of geographic origins, the handling of introduced species, and the distinction between morphological and functional logic in the Cài Bù. L $\overset{ˇ}{ı}$ Shízhēn’s five-class system is shown to be a principled, internally consistent taxonomic framework that, while not phylogenetic, remains coherent within the functional and culinary ontology of its time.

## 1 Introduction

The *Běncǎo Gāngmù* (Compendium of Materia Medica) is a 52-volume encyclopedia of natural history and medicine completed by Lǐ Shízhēn in 1578 and first published at Nanjing in 1596 (Li, 1596). It catalogs 1,892 medicinal substances across 16 sections (Bù) and 60 categories (Lèi), one of the most systematic attempts at natural classification in the Chinese tradition prior to Linnaean taxonomy. Lǐ Shízhēn was born in 1518 in Qízhōu (Hubei Province) and died in 1593, three years before the publication of the work; the work was inscribed on the UNESCO Memory of the World Register in 2011 (UNESCO, 2011).

Within the *Běncǎo Gāngmù*, the vegetable section (Cài Bù) occupies volumes 26–28 and is one of the largest sections, cataloging 105 edible plant and fungal taxa. Lǐ Shízhēn’s opening statement defines its scope: vegetables are ‘anything edible from grass or wood,’ with leek, chive, mallow, onion, and bean leaf as the canonical Five Vegetables that ‘fulfill’ where the five grains ‘nourish.’ This formulation, drawn from the *Huángdì Nèijīng Sùwèn* (Yellow Emperor’s Inner Classic), positions vegetables not as principal foods but as essential dietary complements—a view that shapes the Cài Bù’s scope.

The German sinologist and medical historian Wén Shùdé (Paul U. Unschuld) has produced a series of influential foundational studies that established much of the modern textual-critical basis for research on Lǐ Shízhēn and the Běncǎo Gāngmù (Unschuld, 1986; 2010); the present study builds on this tradition of scholarship. The five-class structure of the Cài Bù itself has also been noted before: Métailié (2007) already identified the same five-class arrangement and argued, as this study also does, that the Běncǎo Gāngmù’s classification is functional rather than phylogenetic and should be evaluated on its own terms rather than against Linnaean or modern systematic botany. What has not received systematic treatment in the English-language botanical or history-of-science literature is the detailed internal logic of this five-class system. Studies have addressed the *Běncǎo Gāngmù* as a whole from pharmacological (Shi et al., 2022), ethnobotanical (Hu, 2005), and bibliographic (Nappi, 2009) perspectives, but the internal criteria of the five-class vegetable system, its taxonomic apparatus, its handling of anomalous taxa, and the case study of fungal classification presented here—as distinct from the prior identification of the five-class structure itself—has not been the focus of a dedicated study. A recent companion study has examined the Běncǎo Gāngmù’s overall four-tiered hierarchy in relation to Linnaean and Darwinian taxonomy (Tsai, 2026) but did not address the internal structure of the Cài Bù specifically. This paper presents a pedagogical analysis of the Běncǎo Gāngmù vegetable section, drawing on open courseware (OCW) materials developed at National Yang Ming Chiao Tung University (NYCU).

This article examines (1) the historical development of vegetable classification in Chinese natural history from the *Shénnóng Běncǎo Jīng* to the *Běncǎo Gāngmù*; (2) the defining criteria and internal logic of each of the five Cài Bù classes; (3) the taxonomic apparatus employed by Lǐ Shízhēn, including his nomenclature system, treatment of synonymy, documentation of geographic origins, and system of inter-section transfers; (4) the classification of the Zhī'ěr Lèi (fungi), which represents a particularly instructive case of taxonomic reasoning; and (5) the relationship between Lǐ Shízhēn’s functional taxonomy and the morphological-phylogenetic taxonomy that emerged in Europe during the same period.

### 1.1 Aim of the study

The present review has five specific aims. First, this study aims to trace the historical development of vegetable classification from the Shénnóng Běncǎo Jīng (ca. 200 AD) through at least 17 successor pharmacopeias to the Běncǎo Gāngmù, thereby establishing the context for Lǐ Shízhēn’s innovation. Second, it characterizes the defining criterion of each of the five Cài Bù classes, examines how Lǐ Shízhēn resolves boundary and anomalous cases, and distinguishes this functional taxonomy from both its predecessors and the Linnaean system. Third, this study documents the Cài Bù’s taxonomic apparatus—the Shìmíng synonymy method, the Jíjiě multi-source annotation method, and the systematic recording of geographic origins for introduced species. Fourth, this study aims to analyze the classification of the Zhī'ěr Lèi (fungi) as a case study in functional versus ontological taxonomy, given Lǐ Shízhēn’s recognition that fungi “are not grass or tree” yet belong in the vegetable section by virtue of edibility. Fifth, it situates the Cài Bù within a comparative framework spanning the three-grade hierarchy of the Shénnóng Běncǎo Jīng, the Linnaean system, and the current APG IV (Angiosperm Phylogeny Group IV classification) framework, arguing that Lǐ Shízhēn’s five-class system is a principled, internally consistent functional taxonomy meriting recognition in the global history of biological classification.

## 2 Methods

A curriculum analysis was conducted on the Vegetable section (Cài Bù) of the open courseware (OCW) materials developed by the author at the School of Chinese Medicine under the NYCU OCW Program. To support this analysis, a systematic narrative review was performed across PubMed/MEDLINE, Web of Science, Google Scholar, and the Cochrane Library. Primary textual sources included the original Chinese text of the Běncǎo Gāngmù. The 1596 Jinling edition, the earliest printing, prepared by Lǐ Shízhēn’s own family and now surviving in only a handful of copies worldwide, was not directly accessible for this study; the text consulted here is a later Qing-dynasty (Kangxi-era, Li, 1672) reprint held at the National Central Library (Taipei), checked where relevant against the openly available Chinese Text Project transcription, which is based on the Qing Sìkù Quánshū recension rather than the Jinling original.

Modern scientific names were matched to each Cài Bù entry using the Flora of China, the APG IV classification (The Angiosperm Phylogeny Group, 2016), and the current mycological and phycological taxonomic literature cited for each relevant entry. Where a vernacular name could not be mapped unambiguously to a single modern binomial—because the source description underdetermines the taxon or because the modern name itself remains contested—the identification is labeled ‘tentative identification’ (morphological description insufficient to distinguish among congeners) or ‘unresolved/synonymous name’ (the modern binomial itself disputed in the taxonomic literature), and the relevant uncertainty is discussed at the point of use rather than resolved by fiat.

## 3 Chinese vegetable classification before the Běnc $\overset{ˇ}{a}$ o Gāngmù

Chinese classification of edible plants predates the pharmacopeia tradition. The *Zhōulǐ* (Rites of Zhou, ca. 300 BC) distinguishes five dietary categories: grains, animals, vegetables, fruits, and condiments. The *Lǐjì* (Book of Rites) specifies the same five canonical vegetables that Lǐ Shízhēn cites as the Cài Bù's conceptual foundation. Classical texts such as the *Shījīng* (Book of Odes) and *Ěryǎ* (the oldest existing monolingual dictionary in the world, ca. 3rd century BC) also contain botanical descriptions that form an early layer of Chinese systematic natural history.

The first pharmacological classification of vegetables appears in the *Shénnóng Běncǎo Jīng* (Divine Farmer’s Classic of Materia Medica, ca. 200 AD), which organizes 365 substances—including vegetables—into three therapeutic quality grades: upper (non-toxic, taken continuously), middle (mildly toxic or tonic), and lower (potent, used only in disease treatment). The system ignores morphological, ecological, or sensory characters, grouping vegetables, minerals, and animals within the same grades. The *Shénnóng Běncǎo Jīng*’s Cài Bù listed 65 vegetable taxa.

Between the *Shénnóng Běncǎo Jīng* and the *Běncǎo Gāngmù*, at least 17 major pharmacopeias updated the vegetable catalog. The most important for the Cài Bù are as follows: the *Míng Yī Bié Lù* (ca. 500 AD, Táo Hóngjǐng), which added 365 new substances; the *Xīnxiū Běncǎo* (659 AD), the first state-sponsored Tang pharmacopeia; the *Běncǎo Shíyì* (741 AD, Chén Cángqì), notable for documenting folk taxa; the *Shíliáo Běncǎo* (early 8th century, Mèng Shēn and Zhāng Dǐng), addressing the dietary properties of edible plants; and the *Jiāyòu Běncǎo* (1060 AD, Zhāng Yǔxī), which added culinary herbs including basil and sweet chrysanthemum. None of these works replaced the three-grade quality hierarchy specifically as the organizing principle for the vegetable catalog, although the broader three-grade framework persisted, in varying degrees, in the overall chapter architecture of several of these texts (Unschuld, 2010).

Lǐ Shízhēn’s major contribution was to synthesize more than 800 prior works—among which specialized pharmacopeias formed only part of the total, alongside literary, philological, and historical works, only a subset of which bear directly on the classificatory question examined here—explicitly citing their disagreements, and to replace the quality-grade system with a functional-morphological classification he judged more useful for medical and dietary purposes. His stated reason was practical: the common people used these plants daily without understanding their distinct properties, so he grouped 105 edible taxa for the Cài Bù into five classes—Hūnxīn, Róuhuá, Luǒcài, Shuǐcài, and Zhī'ěr (Figure 1).

**FIGURE 1:**
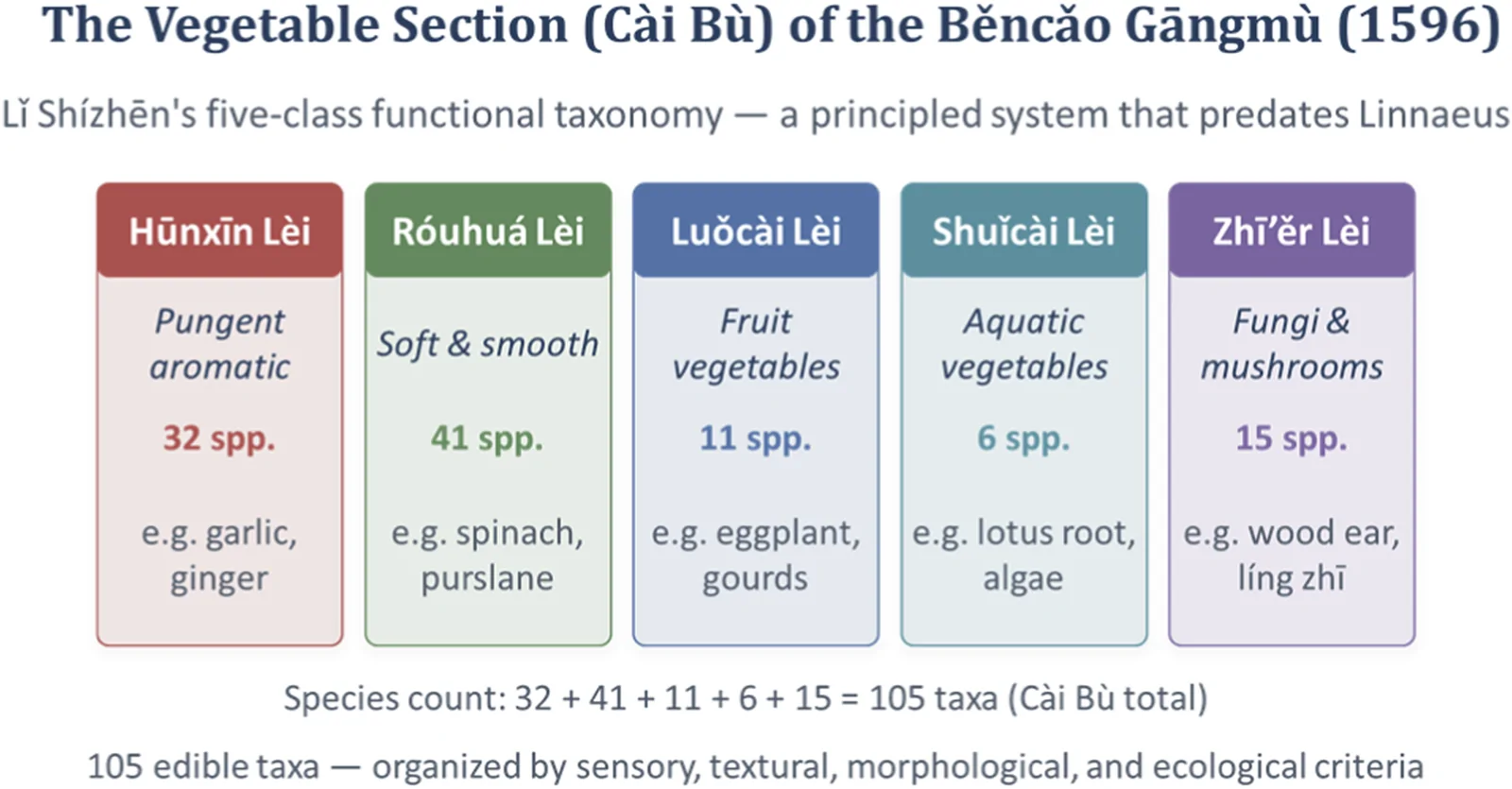
Five-class functional taxonomy of the vegetable section (Cài Bù, Volumes 26–28) of L $\overset{ˇ}{ı}$ Shízhēn’s Běnc $\overset{ˇ}{a}$ o Gāngmù (1596). The five classes—Hūnxīn Lèi (pungent aromatic, 32 taxa), Róuhuá Lèi (soft and smooth, 41 taxa), Lu $\overset{ˇ}{o}$ cài Lèi (fruit vegetables, 11 taxa), Shu $\overset{ˇ}{ı}$ cài Lèi (aquatic vegetables, 6 taxa), and Zhī'ěr Lèi (fungi and mushrooms, 15 taxa)—together comprise the Cài Bù's 105 edible taxa.

## 4 Five-class system: criteria and overall logic

Lǐ Shízhēn’s five-class system for the Cài Bù is a compound classification integrating at least four independent criteria: sensory character, texture, growth form, and ecological habitat. Unlike the Linnaean system—which Lǐ Shízhēn could not have known, since Linnaeus (1707–1778) was born 189 years after Li—the Cài Bù taxonomy does not aspire to reflect natural affinity or evolutionary relationship. It is instead a functional taxonomy that organizes species by their culinary and dietary characteristics to guide preparation, combination, and use. Table 1 summarizes the five classes.

**TABLE 1:** Five-class taxonomy of the Cài Bù in the *Běnc $\overset{ˇ}{a}$ o Gāngmù*.

Class (Pinyin)	Meaning	Volume(s)	Taxa	Defining character	Genus (scientific name/family/common name)
Hūnxīn Lèi	Pungent aromatic	Vol. 26	32	Strong aroma; pungent or acrid taste; warm-natured	Allium (*Allium cepa* L./Amaryllidaceae/Onion), Zingiber (*Zingiber officinale* Roscoe/Zingiberaceae/Ginger)
Róuhuá Lèi	Soft and smooth	Vol. 27	41	Tender, mucilaginous, or smooth texture; mild flavor	Spinacia (*Spinacia oleracea* L./Amaranthaceae/Spinach), Capsella (*Capsella bursa-pastoris* (L.) Medik./Brassicaceae/Shepherd’s purse)
Luǒcài Lèi	Gourd/fruit veg	Vol. 28	11	Edible fruit (gourd, berry, or pepo); vine-grown or bushy	Solanum (*Solanum melongena* L./Solanaceae/Eggplant), Benincasa (*Benincasa hispida* (Thunb.) Cogn./Cucurbitaceae/winter melon)
Shuǐcài Lèi	Aquatic vegetables	Vol. 28	6	Growth in fresh or salt water; algal or aquatic herb	Pyropia (*Pyropia haitanensis*/Bangiaceae/Purple laver), Ulva (*Ulva lactuca* L./Ulvaceae/Sea lettuce)
Zhī'ěr Lèi	Fungi and mushrooms	Vol. 28	15	Fungal growth form; no true leaves, stems, or roots	Ganoderma (*Ganoderma lucidum* (Curtis) P.Karst./Ganodermataceae/Ling zhi; cultivated material = G. sichuanense), auricularia (*Auricularia heimuer* F.Wu, B.K.Cui and Y.C.Dai/Auriculariaceae/Wood ear)

BGM, Běncǎo Gāngmù. APG IV, Angiosperm Phylogeny Group IV classification.

These criteria map differently onto each class: sensory character (pungency, aroma, and volatile compounds) defines the Hūnxīn Lèi; texture and growth form (soft, mucilaginous, or smooth leafy vegetables) defines the Róuhuá Lèi; edible-organ morphology (fleshy fruits borne on vining or bushy plants) defines the Luǒcài Lèi; ecological habitat (aquatic environments) defines the Shuǐcài Lèi; and growth form in the broadest sense (fungal, lacking true leaves, stems, or roots) defines the Zhī'ěr Lèi (Figure 2).

**FIGURE 2:**
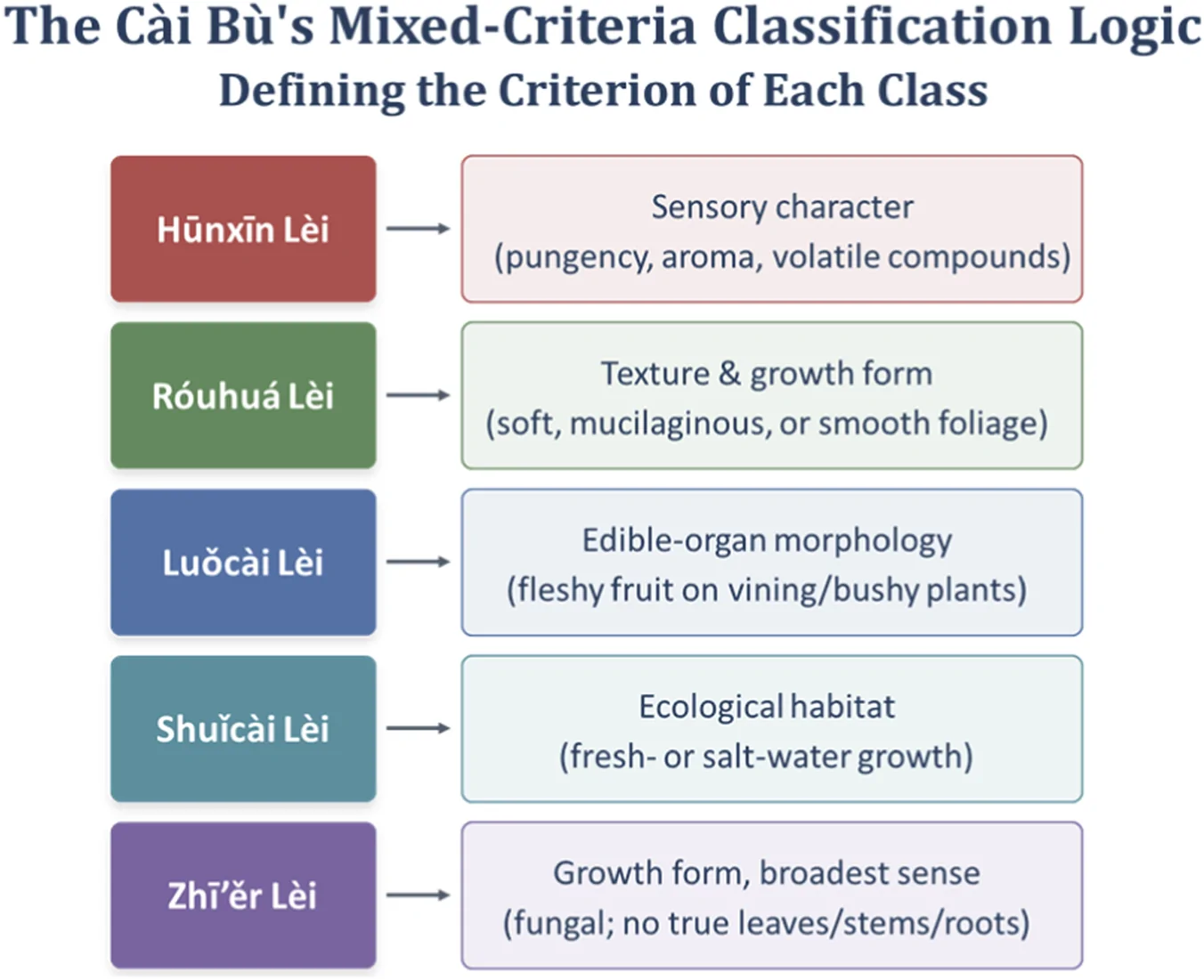
Cài Bù's mixed-criteria classification logic. The single primary criterion defining each of the five classes: sensory character (Hūnxīn Lèi), texture and growth form (Róuhuá Lèi), edible-organ morphology (Luǒcài Lèi), ecological habitat (Shuǐcài Lèi), and growth form in the broadest sense—fungal, with no true leaves, stems, or roots (Zhī'ěr Lèi).

This mixed-criteria approach reflects a deliberate choice to group taxa as a cook, farmer, or physician would, rather than as a morphologist would. The result cuts across what are now very different plant families: the Hūnxīn Lèi groups Amaryllidaceae, Zingiberaceae, Brassicaceae, Apiaceae, Asteraceae, and Lamiaceae, whose members are united solely by their shared aromatic-pungent character, despite having no close phylogenetic relationship.

The five-class system also structurally breaks with all prior classifications by abandoning the tripartite quality hierarchy: all 105 taxa are coequal members of their functional classes, with flavor, thermal nature, and toxicity instead recorded in the Qìwèi (properties and flavor) subsection of each entry. This decoupling of classification from quality ranking was a significant methodological innovation.

### 4.1 Class 1: Hūnxīn Lèi (pungent aromatic vegetables, 32 taxa, volume 26)

The Hūnxīn Lèi is the first class presented in the Cài Bù (the Róuhuá Lèi, with 41 taxa, is the largest by count; Section 4.2) and the only one defined primarily by a sensory-chemical criterion. The terms ‘hūn’ (fragrant, overpoweringly aromatic) and ‘xīn’ (acrid or pungent in taste) together describe the volatile, sulfurous, or terpenoid-rich profile Lǐ Shízhēn associates with this class. He notes that its members are among the ‘Five Pungent Plants (Wǔ xīn)’ of Buddhist dietary restriction, alongside garlic, shallot, leek, onion, and asafoetida—the defining character being the presence of volatile pungent compounds detectable by both smell and taste.

The class encompasses species from several morphologically unrelated plant families, unified by sensory character: (1) the bulb-forming alliums (jiǔ/*Allium tuberosum* Rottler ex Spreng., xie/*A. chinense* G. Don, hú cōng/*A. cepa* L (Aggregatum Group; syn. A. ascalonicum, unresolved name), suan/*A. sativum* L.) from Amaryllidaceae; (2) the rhizomatous ginger (shēng jiāng/*Zingiber officinale* Roscoe) from Zingiberaceae; (3) the taproot vegetables radish (luóbo/*Raphanus sativus* L. (Brassicaceae)) and carrot (hú luóbo/*Daucus carota* L. (Apiaceae)); and (4) the aromatic leafy herbs garland chrysanthemum (tóng hāo/*Glebionis coronaria* (L.) Cass. ex Spach (Asteraceae)) and sweet basil (luó lè/*Ocimum basilicum* L (Lamiaceae)). Table 2 lists the major entries of Hūnxīn Lèi.

**TABLE 2:** Major species of the Hūnxīn Lèi with modern botanical equivalents and classification rationale.

Pinyin name	Modern binomial	Family (APG IV)	Earliest record	Classification rationale in BGM
Jiǔ	*Allium tuberosum* Rottler ex spreng.	Amaryllidaceae	Míng Yī Bié Lù (ca. 500 AD)	Pungent leaf; strong sulfurous aroma
Xiè	*Allium chinense* G.Don	Amaryllidaceae	Míng Yī Bié Lù (ca. 500 AD)	Pungent bulb; one of the canonical five vegetables
Hú cōng	*Allium cepa* L. (aggregatum group; syn. A. ascalonicum auct., an unresolved name)	Amaryllidaceae	Kāibǎo Běncǎo (973)	Pungent bulb; imported from Central asia
Suàn	*Allium sativum* L.	Amaryllidaceae	Míng Yī Bié Lù (ca. 500 AD)	Strongest pungency; one of the ‘five pungent plants'
Láibo	*Raphanus sativus* L.	Brassicaceae	Táng Běncǎo (7th c.)	Pungent root and seed; acrid taste
Shēng jiāng	*Zingiber officinale* Roscoe	Zingiberaceae	Míng Yī Bié Lù (ca. 500 AD)	Pungent rhizome; fresh form, used to disperse cold and stop vomiting
Gān jiāng	*Zingiber officinale* Roscoe	Zingiberaceae	Shénnóng Běncǎo Jīng (ca. 200 AD)	Pungent rhizome; dried form, used to warm the interior and expel deep cold
Tóng hāo	*Glebionis coronaria* (L.) Cass. ex spach	Asteraceae	Jiāyòu Běncǎo (1060)	Aromatic leaf; mild pungency; volatile oils
Hú luóbo	*Daucus carota* L.	Apiaceae	Běncǎo Gāngmù (1596)	Aromatic root; introduced from the Yuan dynasty western trade
Luó lè	*Ocimum basilicum* L.	Lamiaceae	Jiāyòu Běncǎo (1060)	Strongly aromatic leaf; pungent essential oils

Family assignments follow APG IV. ‘Earliest record’ refers to the earliest Chinese pharmacopeia or text in which the taxon appears. BGM, Běncǎo Gāngmù (1596).

Within the Hūnxīn Lèi, Lǐ Shízhēn distinguishes species that are primarily aromatic (hūn), emitting a penetrating odor (alliums and ginger), from those that are primarily pungent (xīn) in taste but lack a strong aroma (radish and relatives). He places radish and carrot here despite their milder aroma because their seeds and root flesh are distinctly acrid raw—a deliberate weighing of taste over smell in borderline cases.

### 4.2 Class 2: Róuhuá Lèi (soft and smooth vegetables, 41 taxa, volume 27)

The Róuhuá Lèi is the largest class in the Cài Bù by species count and the most botanically diverse. The defining character—‘róu’ (soft, tender) and ‘huá’ (smooth, mucilaginous)—is a textural criterion referring both to cooking quality (typically leaves and young stems) and, in many species, to the mucilaginous polysaccharides that give a slippery mouthfeel. This includes spinach (bō léng/*Spinacia oleracea* L. (Amaranthaceae)), purslane (mǎ chǐ xiàn/*Portulaca oleracea* L. (Portulacaceae)), dandelion (pú gōng yīng/*Taraxacum officinale* F.H.Wigg. (Asteraceae)), fish mint (jī/*Houttuynia cordata* Thunb. (Saururaceae)), lily bulb (bǎi hé/*Lilium lancifolium* Thunb. (Liliaceae)), and bamboo shoots (zhú sǔn/*Phyllostachys* spp. and Bambusa spp. (Poaceae))—zhú sǔn functions as a culinary collective category spanning multiple bamboo genera in both the Ming dynasty and modern context, rather than a single species.

The textural criterion has two sub-dimensions. ‘Róu’ (soft/tender) refers to the vegetative growth form: leafy, herbaceous plants with soft cell walls, harvested young and generally eaten lightly cooked or raw. ‘Huá’ (smooth/mucilaginous) refers to taxa with notably high mucilage content—prominently purslane, mallow (kuí/*Malva verticillata* L. (Malvaceae)), and water shield (chún/*Brasenia schreberi* J.F.Gmel. (Cabombaceae))—in which slipperiness is a defining culinary and therapeutic characteristic. The ‘huá’ quality also corresponds to a therapeutic effect recorded in several entries’ Qìwèi (properties and flavor) subsections—a lubricating action on the intestinal tract associated with relief of constipation and diarrhea, a property particularly emphasized for dandelion (pú gōng yīng). The water shield is invoked here only to illustrate the mucilaginous ‘huá' character; its formal classification (Table 1) places it in the Shuǐcài Lèi based on aquatic habitat, which Lǐ Shízhēn treats as taking priority over texture for aquatic taxa. Lǐ Shízhēn notes that purslane’s leaves resemble a horse’s teeth and its texture is ‘slippery and smooth like amaranth, hence the name’ —an etymology linking leaf shape to the textural character, placing it in the Róuhuá Lèi.

### 4.3 Class 3: Lu $\overset{ˇ}{o}$ cài Lèi (gourd and fruit vegetables, 11 taxa, volume 28)

The Luǒcài Lèi (fruit vegetables) is defined morphologically: the edible organ is a fleshy fruit rather than a leaf, stem, root, or bulb. ‘Luǒ’ is an archaic character for large fleshy fruits borne on vining plants, cognate with modern usage for gourd or melon types. The class encompasses eleven taxa: two Solanaceae, eggplant (qié/*Solanum melongena* L.) and bitter eggplant (kǔ qié/*Solanum incanum* L.), and nine Cucurbitaceae, including winter melon (dōng guā/*Benincasa hispida* (Thunb.) Cogn. (Cucurbitaceae)), pumpkin (nán guā/*Cucurbita moschata* Duchesne (Cucurbitaceae)), cucumber (hú guā/*Cucumis sativus* L. (Cucurbitaceae)), loofah (sī guā/*Luffa aegyptiaca* Mill. (Cucurbitaceae)), bitter melon (kǔ guā/*Momordica charantia* L. (Cucurbitaceae)), bottle gourd (hú lú/*Lagenaria siceraria* (Molina) Standl. (Cucurbitaceae)), melon (tián guā/*Cucumis melo* L.), watermelon (xī guā/*Citrullus lanatus* (Thunb.) Matsum. & Nakai), and pickling melon (yuè guā/*Cucumis melo* var. *conomon* (Thunb.) Makino).

Eggplant’s placement in the Luǒcài Lèi rather than the Róuhuá Lèi is an instructive boundary case: its flesh is soft when cooked, which might suggest the Róuhuá Lèi, but Lǐ Shízhēn assigns it by the fruit-type criterion—it is undeniably a fleshy fruit on a bushy plant. He records purple, yellow, white, and blue-water varieties, the last found only in northern regions—varietal documentation revealing awareness of infra-specific diversity beyond the basic species entry.

The pumpkin entry (nán guā/*Cucurbita moschata*) is one of the most recently documented Cài Bù taxa, first appearing in the *Běncǎo Gāngmù* itself. Lǐ Shízhēn records that it originated in southern foreign regions and spread through Fujian and Zhejiang to the Beijing area—‘south’ referring to Southeast Asia, which is consistent with the historically documented introduction of *Cucurbita* species to Asia via Portuguese trade routes in the early 16th century. This historical account of timing and route is a separate matter from the genetic evidence: modern population-genomic analysis of global *C. moschata* germplasm resolves three geographically structured subpopulations, East Asian, African, and American—with the East Asian group genetically distinct from the American source populations (Lee et al., 2021), a finding consistent with, but not itself confirmation of, the specific 16th-century introduction route described above.

### 4.4 Class 4: Shu $\overset{ˇ}{ı}$ cài Lèi (aquatic vegetables, 6 taxa, volume 28)

The Shuǐcài Lèi is the smallest class in the Cài Bù and the only one defined exclusively by ecological habitat: all taxa grow in fresh or salt water, including two marine macroalgae (zǐ cài/*Pyropia* spp., notably P. haitanensis and P. yezoensis (Bangiaceae)—commonly but inaccurately identified with the North Atlantic Porphyra umbilicalis (L.) J. Agardh, which is not cultivated in China, shí chún/*Ulva lactuca* L. (Ulvaceae)), one red alga (lóng xū cài/*Caloglossa leprieurii* (Montagne) G. Martens (Delesseriaceae; tentative identification)), and three aquatic angiosperms: water shield (chún/*Brasenia schreberi* J.F.Gmel. (Cabombaceae)), water chestnut (líng/*Trapa natans* L. (Lythraceae; syn. T. bicornis, an unresolved/synonymous name)), and lotus root (ǒu/*Nelumbo nucifera* Gaertn (Nelumbonaceae)—though the last is also treated partly in the Fruit section).

The ecological criterion creates a heterogeneous class: Rhodophyta, Chlorophyta, Cabombaceae, Lythraceae, and Trapaceae are grouped solely based on aquatic habit. Lǐ Shízhēn is untroubled by this, noting simply that ‘these are vegetables that grow in water and can be eaten.’ The grouping reflects pragmatic classificatory realism: shared harvest methods, preparation requirements, and overlapping TCM therapeutic properties make this a meaningful functional grouping despite the absence of phylogenetic coherence.

#### 4.4.1 Marine algae in a Chinese pharmacopeia

The inclusion of marine algae—zǐ cài (*Pyropia*) and shí chún (*Ulva*)—in the Shuǐcài Lèi is taxonomically significant. Earlier pharmacopeias had scattered algal entries across the Herb section without systematic grouping. Lǐ Shízhēn’s consolidation of algae into the Shuǐcài Lèi, based on aquatic habitat, is among the earliest documented systematic treatments of marine algae as a distinct dietary-ecological category in the Chinese natural history tradition. His Jíjiě for zǐ cài cites Zhū Dānxī’s commentary that ‘all persons with goiter and scrofula should eat purple laver (zǐ cài) regularly, by virtue of the salty flavor’s principle of softening hardness’—embedding a therapeutic observation within what is formally a classification entry, illustrating the inseparability of taxonomy and therapeutics in the Cài Bù.

### 4.5 Class 5: Zhī'ěr Lèi (fungi and mushrooms, 15 taxa, volume 28)

The Zhī'ěr Lèi is the most taxonomically anomalous class in the Cài Bù, and the one whose classification rationale is most explicitly argued by Lǐ Shízhēn. All 15 constituent taxa are fungi—including líng zhī (*Ganoderma lucidum* (Curtis) P. Karst. (Ganodermataceae); cultivated Chinese ling zhi is now assigned to the distinct species G. sichuanense J.D.Zhao & X.Q.Zhang (Wang et al., 2012; Du et al., 2023)), wood ear (mù ěr/*Auricularia heimuer* F. Wu, B.K.Cui, and Y.C.Dai (Auriculariaceae; corrected from A. auricula-judae, a European species not occurring in East Asia, per molecular species clarification by Wu et al., 2014)), stone ear (shí ěr/*Umbilicaria esculenta* (Miyoshi) Minks (Umbilicariaceae)), white jelly fungus (yín ěr/*Tremella fuciformis* Berk (Tremellaceae)), and several polypores. Strictly speaking, shí ěr is not a fungus alone but a lichenized fungus—a stable symbiosis of fungus and alga—which makes the class more heterogeneous, by modern taxonomic standards, than a simple ‘all fungi’ description would suggest. Fungi are not plants. They lack the true leaves, stems, and roots that define the botanical subjects of the other four classes. Their inclusion in the Cài Bù represents a deliberate, reasoned classificatory decision by Lǐ Shízhēn that warrants detailed examination.

In earlier pharmacopeias, edible fungi were distributed across multiple sections based on their substrate or growth habit: tree-growing fungi (including wood ear and líng zhī) were classified in the Wood section (Mù Bù); stone-growing fungi (stone ear) were classified in the Stone section (Shí Bù); and ground-growing fungi were placed in the Herb or Earth sections. Lǐ Shízhēn explicitly rejects this substrate-based classification: ‘The líng zhī mushroom (zhi) is a fungal species. Because fungi can be eaten, they are moved into the vegetable section.’ He then provides the critical qualification: ‘It is a fungal type and belongs among those that can be eaten; therefore, it is entered in the vegetable section.’ The criterion is edibility, not botanical affinity or substrate. Within this class, Lǐ Shízhēn’s differentiation among the five color-coded líng zhī entries also draws on a framework beyond natural observation alone: each color is associated with one of the Five Elements (Wǔ Xíng) and a corresponding organ affinity, integrating Taoist and Yin-Yang cosmology with the empirical criteria of habitat and morphology that otherwise organize the class.

#### 4.5.1 Classification of fungi: a prescient ecological argument

Lǐ Shízhēn’s decision to classify fungi within the vegetable section (Cài Bù)—despite their lacking typical plant traits—is a notable milestone in the history of biological classification. By prioritizing edibility over morphology, his functional taxonomy anticipates the modern concept of a “food taxon.” Contemporary nutrition, food law, and culinary tradition likewise group fungi with vegetables on functional grounds, independent of kingdom boundaries—a distinction formalized only in the mid-20th century (Whittaker, 1969; see also Naranjo-Ortiz and Gabaldón, 2019; Woese et al., 1990).

Lǐ Shízhēn was evidently aware that fungi differ fundamentally from vascular plants. In the Shìmíng (name-derivation) section of the wood ear (mù ěr) entry, he notes, ‘Wood ear (mù ěr) grows on rotten wood; it is not a grass or tree but belongs to the fungal class (Jūn shǔ).’ The explicit recognition that fungi are a separate category—‘not grass or tree’—while still including them in the vegetable section based on edibility reveals a clear-sighted pragmatism: Lǐ Shízhēn’s taxonomy is functional, not ontological. He is classifying ‘things one eats at the table’ (cài), not ‘plants’ in a strict botanical sense. This distinction makes the Cài Bù unusual among pre-modern natural history classifications in that it operates explicitly within a defined scope (edibility) rather than attempting to classify all organisms by their fundamental nature.

## 5 Taxonomic apparatus: nomenclature, synonymy, and etymology

The *Běncǎo Gāngmù*'s taxonomic apparatus is structured around six subsections that appear within each major entry. Understanding these subsections is essential to interpreting Lǐ Shízhēn’s classification practice, because much of the taxonomic reasoning is embedded within them rather than in a separate classificatory statement.

### 5.1 Shìmíng (name derivation)

The Shìmíng subsection—literally ‘releasing the name’ or ‘name explanation’—is Lǐ Shízhēn’s primary vehicle for synonymy and nomenclatural reasoning. For each taxon, he lists all known synonyms, regional variants, and popular names from prior sources, citing the works in which each variant appears. He then provides an etymology for the primary name. Regarding jiǔ (Allium tuberosum), he cites Xǔ Shèn’s Shuōwén Jiězì (ca. 100 AD): ‘The character for jiǔ depicts leaves emerging from the ground. It grows from a single plant for a long time, hence the name jiǔ [phonetically similar to ‘long-lasting’].’ Lǐ Shízhēn adds his own horticultural observation: it can be cut three or four times per year without damaging the root; in winter, it is mounded and covered, and it regrows with the spring—truly a long-lasting plant. The “Shìmíng” thus serves as an etymological dictionary, a list of synonyms, and a morphological and phonological description. This method has limitations: in some entries, Lǐ Shízhēn’s etymological reasoning proceeds by literal, word-by-word interpretation of textual records rather than direct observation of the organism, inferring morphological character from documentary description alone—a caveat worth bearing in mind when using the Shìmíng as evidence of first-hand botanical knowledge.

### 5.2 Jíjiě (comprehensive annotations)

The Jíjiě subsection compiles descriptions and observations from all prior authorities, identified by name and source, that Lǐ Shízhēn judges relevant to the taxon’s identification and classification. Disagreements between authorities are explicitly flagged: the standard formula is ‘X says.; Y says.; I, Shízhēn, now examine.’ This systematic citation of conflicting accounts and Lǐ Shízhēn’s own resolution of them constitutes a proto-critical taxonomic literature review. For the garland chrysanthemum (tóng hāo/*Glebionis coronaria*), he notes, ‘This herb has not been described in the materia medica as to its form; later scholars have not been able to identify it. I, Shízhēn, now describe: it is planted in the eighth and ninth months; in winter and spring, the fat stems are harvested. Flowers and leaves slightly resemble white mugwort; the flavor is pungent-sweet with a wormwood aroma.’ This is a first-person morphological description added to correct an omission in the prior literature—a practice that distinguishes the *Běncǎo Gāngmù* from its predecessors.

### 5.3 Geographic and cultural documentation

A distinctive feature of Lǐ Shízhēn’s taxonomic practice is the systematic documentation of geographic origin, distribution, and cultural context for each taxon. Introduced species are frequently flagged with the prefixes ‘hú’ and ‘fān,’ both broad and often overlapping designations for non-native, exogenous origin in Ming-era usage rather than a systematic Central-Asia/Southeast-Asia distinction, alongside explicit statements of origin in the Jíjiě. The spinach entry (bō léng/*Spinacia oleracea*) provides an exceptionally detailed account: citing the *Táng Huìyào* (Institutional History of the Tang Dynasty), Lǐ Shízhēn records that ‘during the reign of Emperor Taizong, the kingdom of Nipolo [Nepal] presented the spinach (bō léng) vegetable—resembling safflower (hong lan), with fruits such as water caltrops—and when cooked it improves the flavor of food.’ The basil entry (luó lè/*Ocimum basilicum*) records that ‘during the period of Emperor Shi [of the Later Zhao Dynasty, 4th century], the taboo on the word ‘le’ [part of his name] caused people to rename luó lè as ‘fragrant vegetable’ (Xiang cài).’ This cultural–historical annotation, embedded within a taxonomic entry, has few parallels in Linnaean or post-Linnaean botanical classification and represents a systematic effort to document the social and political history of plant names.

## 6 Cài Bù classification in comparative perspective

Lǐ Shízhēn’s five-class taxonomy of the Cài Bù can be compared with three reference systems: the prior Chinese quality-grade classification, the contemporaneous European approach to plant classification, and modern phylogenetic systematics (APG IV; The Angiosperm Phylogeny Group, 2016) (Table 3).

**TABLE 3:** Comparative overview of the three classification frameworks applied to edible plants.

Feature	Shénnóng Běnc $\overset{ˇ}{a}$ o Jīng (ca. 200 AD)	Běncǎo Gāngmù, Cài Bù (1596)	Linnaean system (1753 onward); later phylogenetic revisions noted separately
Primary criterion	Medicinal grade (upper/middle/lower)	Sensory-morphological class (5 categories)	Morphological affinity; later phylogeny
Scope	65 vegetable taxa across three grades	105 taxa across five functional classes	All organisms, unbounded
Fungi treatment	Scattered in multiple sections by medicinal grade	Consolidated into Zhī'ěr Lèi within Cài Bù by edibility	Not distinguished by Linnaeus; recognized as a separate kingdom only later, under Whittaker’s five-kingdom system (1969)
Algae treatment	Not systematically covered	Consolidated into Shuǐcài Lèi by aquatic habitat	Not distinguished by Linnaeus; later split into separate lineages (e.g., rhodophyta and chlorophyta) under modern phylogenetic systematics
Nomenclature	Single canonical name; minimal synonymy	Full synonymy (shìmíng); etymology; regional variants	Binomial nomenclature; author citation; type specimens
Geographic origin	Rarely documented	Systematically noted (e.g., ‘introduced from Hu (western) territory')	Recorded in herbarium data; not part of formal taxonomy
Intersection transfers	Not documented	Explicitly recorded (in/out counts)	Synonymy list; basionym citation

APG IV, angiosperm phylogeny group system, current as of the fourth revision. Linnaean columns refer to the system as it existed after the publication of Species Plantarum (1753).

The most instructive comparison is with Linnaean taxonomy, which was being developed in Europe during the century and a half following Lǐ Shízhēn’s death. Both systems were responding to a perceived inadequacy in prior classification: for Lǐ Shízhēn, the inadequacy of the three-grade quality hierarchy; for Linnaeus, the inadequacy of the pre-existing herbalist and scholastic traditions. Both introduced new, internally consistent criteria: Lǐ Shízhēn based his classification on sensory, morphological, and ecological function, while Linnaeus initially relied on stamens and pistils as the primary criterion (later replaced by overall morphological affinity and, ultimately, by phylogeny). Both systems represented major advances in organizing biological knowledge, and both had limitations that successors corrected.

The most significant limitation of Lǐ Shízhēn’s system, from a modern perspective, is its failure to distinguish phylogenetically related groups. The Hūnxīn Lèi groups Amaryllidaceae, Zingiberaceae, Brassicaceae, Apiaceae, Asteraceae, and Lamiaceae; the Róuhuá Lèi groups Amaranthaceae, Portulacaceae, Asteraceae, Saururaceae, Liliaceae, and Poaceae (bamboo). Within each class, however, the constituent species share the defined sensory or morphological character, and the classes are internally coherent by their own criteria. The system does not claim phylogenetic coherence; it claims functional coherence. Evaluated by its own stated criterion, it is largely successful.

Where Lǐ Shízhēn’s classification most closely approaches modern systematic thinking is in its treatment of the Zhī'ěr Lèi. By explicitly arguing that fungi are neither grass nor trees and belong to a separate category, while simultaneously placing them in the vegetable section on grounds of edibility, Lǐ Shízhēn essentially invents a distinction between ontological taxonomy (what something is) and functional taxonomy (how it is used). This distinction is implicit in modern food science, where ‘vegetable’ is a culinary category that cuts across botanical kingdoms, but it was not articulated as a principle of classification in European natural history until the 19th century. The comparison drawn here between Lǐ Shízhēn’s 1596 argument and this later European distinction is offered as a retrospective analytical parallel, not as a claim that Lǐ Shízhēn foresaw or influenced the later development.

## 7 Legacy and influence of the Cài Bù classification

The *Běncǎo Gāngmù*'s five-class vegetable taxonomy was adopted, with modifications, by subsequent Chinese pharmacopeias. The *Běncǎo Gāngmù Shíyí* (Supplement to the Compendium, 1765, Zhào Xuémǐn) added further taxa within Lǐ Shízhēn’s existing five-class framework, largely preserving its structure. The *Zhíwù Míngshí Túkǎo* (Illustrated Manual of Plant Names and Identifications, 1848, Wú Qíjùn), which represented one of China’s most systematic pre-modern botanical works, adopted elements of Lǐ Shízhēn’s nomenclatural practice—particularly the Shìmíng synonymy system—while restructuring the overall classification around morphological criteria closer to Linnaean principles (Wu, 1848).

Western scholars first engaged with the *Běncǎo Gāngmù* classification system through the translations and commentaries of Jesuit missionaries from the 17th century onward. The naturalist Engelbert Kaempfer (1651–1716) drew on *Běncǎo Gāngmù* material in his account of East Asian natural history. The sinologist Emil Bretschneider (1833–1901) published the first systematic attempt to correlate *Běncǎo Gāngmù* taxa with Linnaean binomials (Bretschneider, 1881), a project that identified the Cài Bù as one of the most tractable sections of the work for Western botanical identification, precisely because its species are economically important cultivated vegetables with well-established Latin names.

The modern relevance of Lǐ Shízhēn’s system lies chiefly in its status as a reference framework for ethnobotanical and food-historical research: the five classes still organize categories in traditional Chinese dietary medicine (Shí liáo), and the species list serves as a canonical inventory against which modern cultivar surveys and food-safety regulations can be benchmarked. Integrative approaches that combine Běncǎo textual research with chloroplast genome sequencing and phylogenetic analysis have recently been used to authenticate the botanical identity of other Běncǎo-listed taxa (Sun et al., 2023), illustrating a methodology directly applicable to unresolved Cài Bù identifications, such as the algal and bamboo entries discussed above. The UNESCO inscription of the *Běncǎo Gāngmù* on the Memory of the World Register (documentary-textual heritage) in 2011 has further stimulated scholarly interest in its classification system as a landmark of Chinese natural history (UNESCO, 2011; Simoons, 1991).

## 8 Conclusion

The Cài Bù of Lǐ Shízhēn’s *Běncǎo Gāngmù* constitutes a principled and internally consistent classification of 105 edible plant and fungal taxa, organized into five functional–morphological classes that depart decisively from the three-grade quality hierarchy of all prior Chinese pharmacopeias. The five classes—Hūnxīn Lèi (pungent aromatic), Róuhuá Lèi (soft and smooth), Luǒcài Lèi (fruit vegetables), Shuǐcài Lèi (aquatic vegetables), and Zhī'ěr Lèi (fungi and mushrooms)—are defined by compound criteria that integrate sensory character, texture, growth form, and ecological habitat and are applied with demonstrable consistency across the 105 constituent taxa.

The taxonomic apparatus of the Cài Bù—including its Shìmíng nomenclature system, its Jíjiě multi-source annotation, its systematic documentation of geographic origin, and its explicit accounting of inter-section transfers—represents the most sophisticated framework for organizing biological knowledge in the Chinese tradition prior to the introduction of Linnaean systematics. Lǐ Shízhēn’s treatment of fungi is particularly noteworthy: his explicit recognition that fungi are not grass or trees, while retaining them in the vegetable section on grounds of edibility, constitutes a clear-sighted functional taxonomy that anticipates later distinctions between ontological and culinary classification.

The Cài Bù classification is best understood not as a failed attempt at phylogenetic systematics but as a successful attempt at a different kind of taxonomy, oriented toward dietary, agricultural, and medical practice rather than natural affinity. By this criterion, Lǐ Shízhēn’s five-class system is coherent, well-reasoned, and original, meriting recognition in the global history of biological classification.
